# Effects of armed conflicts on childhood undernutrition in Africa: a systematic review and meta-analysis

**DOI:** 10.1186/s13643-023-02206-4

**Published:** 2023-03-15

**Authors:** Melkalem Mamuye Azanaw, Denekew Tenaw Anley, Rahel Mulatie Anteneh, Getachew Arage, Achenef Asmamaw Muche

**Affiliations:** 1 Department of Public Health, College of Health Sciences, Debre Tabor University, Debre Tabor, Ethiopia; 2 Department of Nutrition and Dietetics, College of Health Sciences, Debre Tabor University, Debre Tabor, Ethiopia; 3 Department of Epidemiology and Biostatics, Institute of Public Health, University of Gondar, Gondar, Ethiopia

## Abstract

**Background:**

Undernutrition is defined as not consuming enough nutrients and energy to meet one’s needs for maintaining good health. It is exacerbated by armed conflict. Individuals cannot stick to jobs because of a lack of safety during conflicts, which has an impact on families’ ability to purchase food. However, there is a paucity of evidence on pooled evidence on the impact of armed conflict on childhood undernutrition among children aged 6 to 59 months in Africa. Therefore, this review aimed to examine the effects of armed conflict on the magnitude of undernutrition, particularly stunting, underweight, and wasting among children in Africa.

**Methods:**

A comprehensive literature search was conducted using electronic databases (PubMed, Hinari, and Google Scholar database) to locate potential studies. Heterogeneity between studies was checked using Cochrane *Q* test statistics and *I*^2^ test statistics. Small-study effects were checked using Egger’s statistical test at a 5% significance level. A random-effects model was employed to estimate the pooled prevalence and associated factors of undernutrition among children aged 6–59 months in Africa.

**Results:**

Of a total of 585 articles retrieved from the databases, 12 studies met our inclusion criteria. The pooled prevalence of wasting, stunting, and being underweight among conflict-affected African countries was 20.25% (95%CI = 15.08–25.43), 34.18% (95% CI = 26.34–42.02), and 24.00% (95%CI = 16.35–31.65), respectively. The most consistent factors associated with childhood stunting, wasting, and being underweight in Africa were low mother’s education, prolonged duration of armed conflict, and rural place of residence.

**Conclusion:**

The severity of malnutrition crises will be assisted by a better understanding of the variables associated with child malnutrition, which will improve the effectiveness of development and humanitarian responses. We urge that health planners, policymakers, and the general public prioritize children with acute malnutrition in Africa’s conflict-affected areas.

**Systematic review registration:**

PROSPERO CRD42022367487

**Supplementary Information:**

The online version contains supplementary material available at 10.1186/s13643-023-02206-4.

## Background

Armed conflict is defined as a political conflict in which at least one state’s armed forces engage in armed conflict and at least 1000 people are killed as a result of the fighting [1]. Armed conflict is a danger to global peace and a barrier to progress toward long-term development. Conflict discourages capital accumulation and undermines production capacities, stifling economic expansion on a global scale [2].

Undernutrition is defined as not consuming enough nutrients and energy to meet one’s needs for maintaining good health. The triple burden of malnutrition will continue to scourge the world’s children’s nutrition in 2020. The first burden is the persistent scourge of undernutrition, which threatens the survival, growth, and development of millions of children and impedes the advancement of economies and nations. Therefore, according to the UNICEF Convention on the Rights of the Child, countries must work toward full implementation of and take the necessary strategies to address malnutrition, including providing enough nutrient-rich food [2].

Undernutrition includes wasting, stunting, being underweight, and inadequate vitamins or minerals [3, 4]. In 2014, the global prevalence of underweight, stunted, and wasted children were estimated to be 14.6, 23.8, and 7%, respectively [5]. In addition, undernutrition among children under five has an increasing trend over the period between 2006 and 2014. For instance, underweight prevalence rose from 27% in 2006 to 29.7% in 2010. By 2014, this rate increased further to 33% [5]. Sub-Saharan Africa (SSA) accounted for one-third of all malnutrition among children worldwide between 1990 and 2015 [6].

Different studies in the world indicate that armed conflict has been associated with childhood malnutrition, and there is a dearth of research into its influence on their nutritional status [6]. The indirect linkage between conflict and children’s nutritional status is influenced by sociodemographic characteristics. Undernutrition is exacerbated by armed conflict. Individuals cannot stick to jobs because of a lack of safety during conflicts, which has an impact on families’ ability to purchase food [7].

Different studies identified associated factors with armed conflict and childhood undernutrition in different regions of the world. Children from low educated [8–10], poor [9], and rural mothers [11]; duration of the conflict [9, 12–14]; male gender; older age [8, 12]; and rural place of residence [9, 14] were positively associated to childhood undernutrition in conflict-affected countries in Africa.

To the best of the investigators’ knowledge, despite the association between armed conflict and poor nutritional status among children has been documented, there is a paucity of evidence on pooled evidence on the impact of armed conflict on childhood undernutrition among children aged 6 to 59 months in Africa. Therefore, this review aimed to examine the effects of armed conflict on the magnitude of undernutrition, particularly stunting, underweight, and wasting among children in Africa. Furthermore, the pooled association between armed conflict and childhood malnutrition will help policymakers, programmers, and funders to focus on aggravating factors during conflict.

## Main text

### Reporting and protocol registration

The protocol used in this study was registered with the International Prospective Register of Systematic Reviews (PROSPERO) and can be accessed with the registration number CRD42022367487.

### Study setting and search strategy

Searches were carried out using PubMed/MEDLINE, Hinari, and Google Scholar databases. Unpublished papers were also checked out of academic libraries and research centers. From February 1 to March 25, 2022, a systematic search of all electronic databases was done. Pre-identified search terms were used to allow a comprehensive search strategy that included all the relevant studies. Pre-identified search terms such as “Armed Conflict” [Mesh] or “war” or “Conflict, Armed” and “Malnutrition” [Mesh]” or “Nutritional Deficiency” or “Nutritional Deficiencies” or “Undernutrition” was used.

### Eligibility criteria

#### Population and condition

Studies reporting the magnitude of the impacts of armed conflict on stunting, underweight, and wasting among children aged 6–59 months in Africa were included.

#### Inclusion setting/context

All studies that were conducted in African countries are included. All included studies were published.

#### Study design

All articles that were conducted by cross-sectional study design are included due to the given nature of focus, armed conflict.

#### Language

Full-text articles written in the English language were included.

#### Publication year

Studies published after the 2015 year of publication were included.

### Exclusion criteria

Low-quality studies were excluded.

### Measurement of the outcome variable

The primary measure of the outcome of this review was the magnitude of childhood undernutrition among countries with armed conflict. The second outcome was determinants of childhood undernutrition in conflict-affected African countries, which were determined using ORs, the mean which is calculated based on binary and continuous outcomes from the included primary studies.

### Study selection and data collection

All the studies reviewed through different electronic databases were combined, exported, and managed using the Endnote version X7.2 (Thomson Reuters, Philadelphia, PA, USA) software. All duplicate studies were removed, and full-text studies were downloaded manually and with the use of the Endnote software. Each study’s eligibility was thoroughly evaluated by two reviewers who worked separately (MM and RM). Then, based on the titles and abstracts, papers were evaluated and rejected. The remaining articles were evaluated for full-text articles or reports. A Preferred Reporting Items for Systematic Reviews and Meta-Analyses (PRISMA) flowchart of the selection process will be included in the systematic review [15].

### Assessment of quality of individual studies

The studies’ quality was assessed using a standard quality assessment tool (hoy tool). There are nine questions with the lowest score that had the least risk of bias. Overall scores range from 0 to 3, 4 to 6, and 7 to 9, indicating a low, moderate, or high risk of bias, respectively [16]. The studies were evaluated by three reviewers who worked separately (MMA, RMA, and DTA). Dissensions between them were settled by a different review team (AA and GA).

### Data extraction and management

Two reviewers (MMA and RMA) separately extracted the data on a Microsoft Excel spreadsheet using a standardized data extraction checklist. Disagreements between the two authors were resolved through conversation and the intervention of another reviewer (DTA). The authors, year of publication, country, study design, sample size, the prevalence of undernutrition with standard error, and determinant factors, as well as effect size and standard error, were extracted for each study.

### Statistical analysis

The data were extracted and then imported into the STATA/MP version 16.0 software for analysis. The random-effects approach with Der Simonian-Laird model weights was used to examine the pooled prevalence of stunting, underweight, and wasting, as well as its associated factors [17]. Statistically, significant heterogeneity was assessed using the checked by Cochrane *Q* test and *I*^2^ statistics [17]. To minimize the variance of estimated points between primary studies, a subgroup analysis was carried out by country. A sensitivity analysis was conducted to determine the influence of single studies on the pooled estimates. Univariate meta-regression was conducted using mean age in the study using a random-effects model. Publication bias (small study effect) was checked using graphically using a funnel plot and Egger’s statistical test [18]. Statistically significant Egger’s test (*P* value < 0.05) indicates the presence of a small study effect and is handled by non-parametric trim and fill analysis using the random-effects model [19].

## Results

### Study selection and identification

**Fig. 1:**
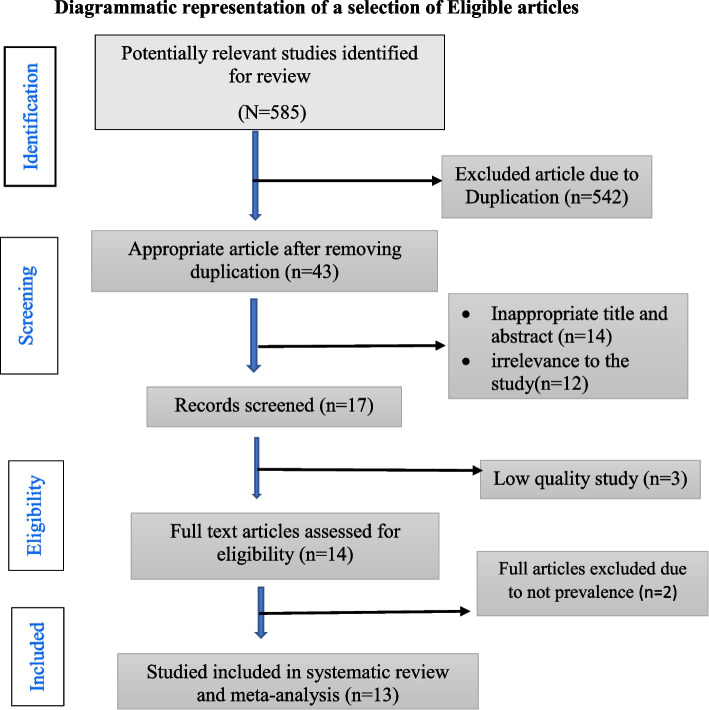
PRISMA flow diagram for the studies screened, reviewed, and included in Africa, 2022

Of the 585 papers searched from different databases, 542 duplicates were removed again and 14 were removed because of irrelevance to the study; again, 12 papers were removed by reading the title and the abstract. Finally, out of the remaining 17 papers, 5 were excluded due to low quality, the outcome not reported, and the year of publication. Finally, 12 papers were used for the meta-analysis (Fig. 1).

### Characteristics of included studies

All the included studies were cross-sectional studies. A total of 140,291 children in the age group 6–59 months were included in the review. The mean age was 29.90 ± 4.36 months. The minimum and the maximum ages were 20 and 35 months, respectively. The minimum (2.3%) and maximum (29.3%) prevalence of wasting among children were reported in South Sudan [12] and Nigeria [20], respectively (Table 1).

**Table 1:** Characteristics of studies included

Authors, years	Country	Region	Design	Quality	Sample size	Effects of armed conflict on childhood nutrition	Factors +vely associated with undernutrition in conflict areas
Kien Le, 2021 [11]	DRC	Central Africa	Surveys	1	10,600	• Makes children weigh less for their age by 0.20 SDS. • Makes children weigh less than their height by 0.24 SDs. • Increases the magnitude underweight by 17.6%. • Increases the magnitude of waste by 35%.	• Low-educated mothers, poor mothers, and rural mothers
Kinyoki DK et al., 2017 [21]	Somalia	East Africa	Surveys	1	73,778	• The effects of the recent and longer-term conflict were large, with 76% and 88% greater odds of wasting and stunting and nationally, attributable fractions of 13% and 14%, respectively.	• Log duration of the conflict
Kountchou et al., 2019 [22]	Chad	North-central Africa	Surveys	1	18,463	• 43% of children are stunted. • 33% of children being underweight. • 15% of children are wasted given the mean value of the weight-forage.	• Male sex • Older age • Low education
Howell, 2020 [14]	Nigeria	West Africa	survey	1	15,961	• Close to a conflict zone and acute malnutrition was high.	• Rural residence • Long duration of the conflict
Samson Olufunminiyi Idowu et al., 2020 [23]	Nigeria	West Africa	Survey	1	288	• Children in conflict zones had significantly undernourished (stunted, wasted, and underweight).	• Association is not conducted
Mulugeta Gebregziabher [24]	Ethiopia	East Africa	Survey	1	3269	• Severe acute malnutrition increased significantly from 1% in 2019 (pre-war) to 6%, and moderate acute malnutrition increased significantly from 8 to 22%. • Global acute malnutrition nearly tripled from 10% in 2019 to 28%.	• Association is not conducted
Dahab et al., 2020 [9]	Sudan	East Africa	Survey	1	14,081	• Armed conflict is associated with a greater risk of severe and moderate underweight. • There is statistical evidence of an association between armed conflict and the risk of severe underweight.	• Low education • Poorest wealth index • Rural residence
Gillian Dunn, 2018 [10]	Nigeria	West Africa	Surveys	1	2572	• Armed conflict decreases wasting	• Low education
Kiarie et al., 2021 [12]	South Sudan	East Africa	Surveys	1	630	• The prevalence of undernutrition explained by wasting, underweight, and stunting were 2.3%, 4.8%, and 23.8%, respectively.	• Male sex • Older age
Sumbele et al., 2020 [25]	Cameroon	West Africa	Surveys	1	649	• Stunting, underweight, and wasting occurred in 31.3, 13.1, and 6.3% of the children, respectively.	• Low educational • Older age

### The pooled prevalence of undernourishment (stunting, wasting, and underweight) in armed conflict areas

**Fig. 2:**
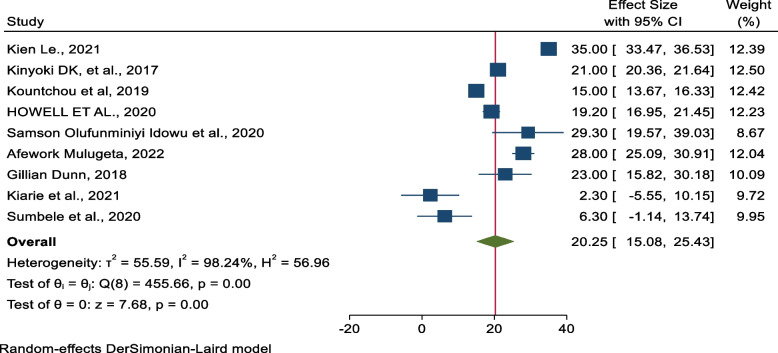
The pooled prevalence of wasting among children aged 6–59 months in conflict-affected countries in Africa

In the random effects model, the pooled prevalence of wasting, stunting, and being underweight was 20.25% (95% CI = 15.08–25.43, *I*^2^ = 98.24, *P* value < 0.001) (Fig. 2), 34.18% (95% CI = 26.34–42.02, *I*^2^ = 98.24, *P* value < 0.001), and 24.00% (95% CI = 16.35–31.65, *I*^2^ = 98.66, *P* value < 0.001), respectively (Supplementary (Supp) Figs. S1 and S2).

### Small study effects

The small study effect (publication bias) was assessed by Egger’s test bias for the prevalence of wasting (supplementary file), stunting, and being underweight. The funnel plot and Egger’s regression tests showed that there is no evidence of substantial publication bias for the prevalence of undernutrition (wasting, stunting, and underweight) among children in Africa (Supp Figs. S3, S4, S5, and S6).

### Handling heterogeneity

**Fig. 3:**
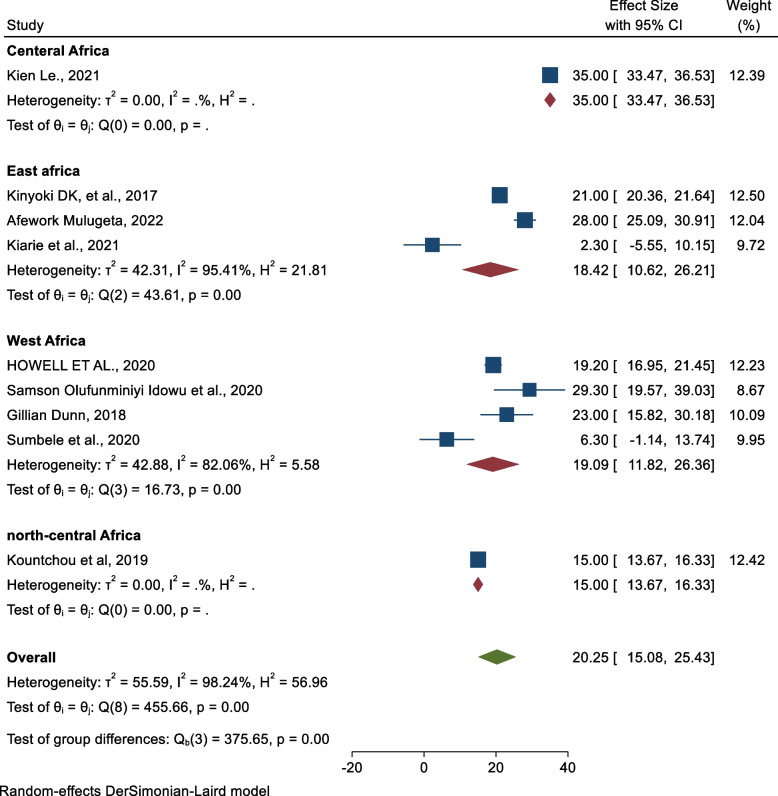
Subgroup analysis by regions for prevalence of wasting among children aged 6–59 months in conflict-affected countries in Africa

Significant heterogeneity was observed from the random-effects model pooled estimate. To handle this heterogeneity, sensitivity analysis, subgroup analysis, and meta-regression analysis were performed. In sensitivity analysis, there were no studies that excessively influence the pooled prevalence of undernutrition (wasting, stunting, and underweight). Subgroup analysis was performed based on the region and age of the child. Based on the region, the highest prevalence of wasting was observed in a study conducted in Central Africa 35%, and the lowest prevalence was observed in the Northcentral Africa region 15.00% (Fig. 3). On the other hand, the highest prevalence of stunting was observed in studies conducted in Northcentral Africa (43%) and the lowest was observed in East Africa (28.28%) (Table 2).

**Table 2:** Subgroup analysis for childhood nutrition and its association with armed conflict in Africa

Variables		Included studies	Prevalence (95%CI)		
			Wasting	Stunting	Underweight
By region	Northcentral Africa	3	15.00 (13.67, 16.33)	43.00 (41.91, 44.08)	33.00 (31.82, 34.18)
	East Africa	3	18.42 (10.62, 26.21)	28.28 (21.44, 35.12)	19.65 (8.96, 48.26)
	Central Africa	1	35.0 (33.47, 36.54)	–	17.00 (15.27, 18.73)
	West Africa	5	19.09 (11.82, 26.36)	36.40 (26.90, 45.89)	27.30 (1.02, 55.61)
By age of child	6–24 months	2	17.58 (4.96, 40.11)	36.34 (26.90, 45.89)	27.30 (–1.02, 55.61)
	25–59 months	9	21.00 (15.32, 26.68)	33.06 (23.24, 42.87)	22.93 (14.07, 31.78)

### Meta-regression

Meta-regression analysis was computed to evaluate underlying sources of heterogeneity using mean age and year of publication. No significant association was observed between undernutrition (wasting, stunting, and underweight) and the above-described variables (Table 3). The linear relationship between mean age and wasting was not diagonal (Supp Fig. S6).

**Table 3:** Univariate meta-regression analysis result for the prevalence of childhood nutrition in armed conflict African countries

Study level variables	Adjusted *R*^2^			Coefficients (95% CI)		
	Wasting	Stunting	Underweight	Wasting	Stunting	Underweight
Mean age	0.00	0.00	0.00	0.53 (–0.92, 1.97)	–0.21 (–2.02, 1.58)	–0.05 (–2.51, 2.39)
Year of publication	0.00	0.00	0.00	0.61 (–3.67, 4.87)	–1.98 (10.83, 6.85)	–12.00 (23.85, 0.15)

*CI* confidence interval

*Statistically significant at 5% level

### Factors associated with childhood undernutrition in armed conflict areas

To identify the association of wasting with educational status, three studies were included [9–11]. The pooled estimate showed a significant association between low education and waste in conflict-affected areas. Children from mothers with low education and armed conflict areas are 12% more likely to have waste as compared with their counterparts (POR = 1.12, 95% CI (1.05, 1.20). There was no small study effect as evidenced by Egger’s test (*P* value = 0.356).

In a meta-analysis of three studies [9, 11, 14], children that reside in rural areas are 1.46 times more likely to have waste as compared to those who reside in urban areas (POR = 1.46, 95% CI 1. 1.04, 2.04). High heterogeneity was observed in the random-effects model (*I*^2^ = 92.3%, *P* value = 0.023). Egger’s test showed no small study effect (*P* value = 0.10).

Two studies [9, 12] were included to assess the pooled association between long-duration of conflict and childhood wasting. The pooled result showed children with a long duration of armed conflict are 2.60 times more likely to have wasting among children (POR = 2.60, 95% CI 1.87, 4.33). High heterogeneity was observed from the random-effects model (*I*^2^= 94.92%, *P* value =0.003). Egger’s test showed no small study effect (*P* value = 0.236), and no study influenced the estimates (Table 4).

**Table 4:** Summary of the pooled effects of factors associated with childhood wasting in conflict-affected areas in Africa, 2022

Variables	POR (95%CI)	Heterogeneity (*I*^2,^ *P* value)	Egger’s *P* value	Total studies
Wealth status				
Rich	1			
Poor	1.42 (0.95, 2.10)	77.67%, 0.085	0.5082	3
Educational status				
High	1			
Low	1.12 (1.05, 1.20)	87.44%, 0.001	0.356	3
Place of residence				
Urban	1			
Rural	1.46 (1.04, 2.04)	92.3%, 0.0274	0.100	3
Duration of conflict				
Short	1			
Long	2.60 (1.87, 4.33)	94.92, 0.003	0.236	2
Sex of child				
Male				
Female	1.22 (0.84, s1.77)	99.97, 0.294	0.7853	3

## Discussion

The purpose of this study was to examine the prevalence of poor nutritional outcomes among children in Africa who were affected by armed conflict, as well as the mechanisms underlying the relationship between armed conflict and undernourishment. Twelve studies from 10 countries were included in the review. This is the first study to study the impact of armed conflict on children’s nutritional status in Africa. It is also the first research to investigate the factors of malnutrition in a situation of armed conflict using pooled estimates.

Our findings reveal a significant increment in the prevalence of malnutrition in armed conflict areas. The overall pooled magnitude of wasting among children was 20.25% (95% CI = 15.08–25.43). The overall pooled magnitude of underweight among children was 24.00% (95% CI = 16.35–31.65). This finding is much lower than studies conducted in the Democratic Republic of Congo (DRC) [11]. This systematic review and meta-analysis also revealed that the pooled prevalence of underweight in conflict-affected areas in Africa was 34.18% (95% CI = 26.34–42.02). This discrepancy might be due to the longer duration of the conflict in DRC whereas the current finding is much higher than the studies conducted in Sudan [9], South Sudan [12], and Nigeria [10, 14, 20, 26]. Individuals are unable to maintain employment due to a lack of safety during disputes, which has a negative influence on families’ ability to purchase food [20].

From subgroup analysis by region, the highest prevalence of wasting (35%) was observed in Central Africa. Furthermore, from subgroup analysis by child age, the highest prevalence of stunting (36.34%) was also observed in the age of a child between 6 and 24 months. The possible justification might be that armed conflict may persist for a long period in central Africa than in other regions and the age group 6 to 24 months is the critical age for child development which is more vulnerable to acute malnutrition [27].

This review also identifies the determinant factors of childhood undernutrition. In the random effects model pooled estimate, maternal education, duration of armed conflict, place of residence, and sex of child were significantly associated with undernourishment in armed conflict areas [2, 10, 12, 14, 20, 21, 28].

From the random effects model estimate, children from mothers with low education and armed conflict areas are 12% more likely to have wasted as compared with their counterparts. This finding is consistent in studies conducted in Sub-Saharan Africa [29].

In a meta-analysis, children that reside in rural areas are 1.46 times more likely to have wasting as compared to those who reside in urban areas. This might be because rural areas are far from service utilization. Furthermore, the finding is that rural children experienced more conflicts than urban children due to most armed conflict is experienced in rural than in towns and cities.

The pooled result showed children with a long duration of armed conflict are 2.60 times more likely to have wasting among children. Our findings are generally consistent with findings from other countries on the negative impact of conflict on child nutrition. However, these results provide an improved methodology for relating the size, duration, and location of a conflict to child malnutrition. Most previous studies have examined conflicts in a particular region of a country but have not distinguished between how close the conflict was to the mother and child’s residence and generally have not examined conflict severity.

The major limitation of this study is the small number of published papers that may have an impact on the results obtained. It is also difficult to conduct meta-regression for the current number of articles to handle heterogeneity.

## Conclusions

In conclusion, armed conflict affects childhood malnutrition in Africa. The highest prevalence of wasting was observed in central Africa and the lowest was in Northcentral Africa. Maternal education, place of residence, and duration of armed conflict were statistically significant factors for wasting. Based on the finding of this review, we recommend that health planners, policymakers, and the community itself should give prior attention to children with acute malnutrition in conflict-affected areas in Africa.

## Supplementary Information

**Additional file 1: Supp fig 1.** Pooled prevalence of stunting among children age 6-59 months in conflict affected countries in Africa. **Supp fig 2.** Pooled prevalence of underweight among children age 6-59 months in conflict affected countries in Africa. **Supp fig 3.** Funnel plot of small study effects for the prevalence of wasting among children age 6-59 months in conflict-affected countries in Africa. **Supp fig 4.** Funnel plot of small study effects for the prevalence of stunting among children age 6-59 months in conflict-affected countries in Africa. **Supp fig 5.** Funnel plot of small study effects for the prevalence of underweight among children age 6-59 months in conflict-affected countries in Africa. **Supp fig 6.** Funnel plot of trill and fill for the prevalence of underweight among children age 6-59 months in conflict-affected countries in Africa. **Supp fig 7.** The relationship between mean age in the month and wasting among children in armed conflict-affected countries in Africa, 2022.

## Data Availability

Data were extracted from published sources.

## Abbreviations

CI: Confidence interval
OR: Odds ratio
POR: Pooled odds ratio
PRISMA: Preferred Reporting Items for Systematic Reviews and Meta-Analyses
SSA: Sub-Saharan Africa
