# Supplementary material for: Effects of armed conflicts on childhood undernutrition in Africa: a systematic review and meta-analysis
Source: Syst Rev. 2023 Mar 15;12:46. doi: 10.1186/s13643-023-02206-4 (PMC10015806; doi:10.1186/s13643-023-02206-4)
Supplement: Supplementary file 1 — Additional file 1: Supp fig 1. Pooled prevalence of stunting among children age 6-59 months in conflict affected countries in Africa. Supp fig 2. Pooled prevalence of underweight among children age 6-59 months in conflict affected countries in Africa. Supp fig 3. Funnel plot of small study effects for the prevalence of wasting among children age 6-59 months in conflict-affected countries in Africa. Supp fig 4. Funnel plot of small study effects for the prevalence of stunting among children age 6-59 months in conflict-affected countries in Africa. Supp fig 5. Funnel plot of small study effects for the prevalence of underweight among children age 6-59 months in conflict-affected countries in Africa. Supp fig 6. Funnel plot of trill and fill for the prevalence of underweight among children age 6-59 months in conflict-affected countries in Africa. Supp fig 7. The relationship between mean age in the month and wasting among children in armed conflict-affected countries in Africa, 2022. [file 13643_2023_2206_MOESM1_ESM.docx]

Supp fig 1. Pooled prevalence of stunting among children age 6-59 months in conflict affected countries in Africa.

Supp fig 2. Pooled prevalence of underweight among children age 6-59 months in conflict affected countries in Africa.

Supp fig 3. Funnel plot of small study effects for the prevalence of wasting among children age 6-59 months in conflict-affected countries in Africa.

Supp fig 4. Funnel plot of small study effects for the prevalence of stunting among children age 6-59 months in conflict-affected countries in Africa.

Supp fig 5. Funnel plot of small study effects for the prevalence of underweight among children age 6-59 months in conflict-affected countries in Africa.

Supp fig 6. Funnel plot of trill and fill for the prevalence of underweight among children age 6-59 months in conflict-affected countries in Africa.

Supp fig7: The relationship between mean age in the month and wasting among children in armed conflict-affected countries in Africa, 2022.
